# Correction: Safety and immunogenicity of fractional COVID-19 vaccine doses in Nigerian adults: A randomized non-inferiority trial

**DOI:** 10.1038/s41598-025-16245-5

**Published:** 2025-08-20

**Authors:** Abideen Salako, Adesola Musa, Fehintola Ige, Adam Abdullahi, Ayorinde James, Sabdat Ekama, Oluwatosin Odubela, Ifeoma Idigbe, Olusola Ajibaye, Mazharul Altaf, Kazeem Adeneye, Folahanmi T. Akinsolu, Ifedola Olojo, Azuka Okwuraiwe, Henry Egharevba, Magaret Ekpenyong, Uchenna Elemuwa, Ifeoma Ezenyi, Fraden Bitrus, Olayemi Odubela, Abdulrasheed Oba, Ganiu Idris, Jimoh Yusuf, Ibukun Akande, Stephine Nwaiwu, Louisa Omale, Oluwatobiloba Oyewunmi, Adedoyin Agbabiaka, Olajumoke Eyinade, Joy Ogunwale, Abdullah Garba, Yahya Bello, Baba Musa, Ogochukwu Ezejiofor, Ben Ejiro, Bamidele Iwalokun, Leah Rosenzweig, Obi Adigwe, Christianah Adeyeye, Faisal Shuaib, Witold Wicek, Yohhei Hamada, Oliver Ezechi, Ravindra Gupta, Babatunde Salako

**Affiliations:** 1 Nigerian Institute of Medical Research, Yaba, Lagos Nigeria; 2 Department of Medicine and Cambridge Institute of Therapeutic Immunology & Infectious Disease (CITIID), University of Cambridge, Cambridge, UK; 3 Takemi Program in International Health, Harvard T.H Chan School of Public Health, Boston, MA USA; 4 National Institute for Pharmaceutical Research, Innovation and Development Abuja Federal Capital Territory, Abuja, Nigeria; 5 National Agency for Food Drug, Administration and Control, Abuja, FCT Nigeria; 6 National Primary Health Development Agency, Abuja, Nigeria; 7 Department of Medicine/Africa Center of Excellence for Population Health and Policy, Bayero University Kano, Kano, Kano State Nigeria; 8 Nnamdi Azikiwe University Teaching Hospital, Nnewi, Enugu State Nigeria; 9 Delta State University Teaching Hospital, Oghara, Delta State Nigeria; 10 Development Innovation Lab, University of Chicago, Chicago, IL USA; 11 Institute for Global Health, University College London, London, UK; 12 Africa Health Research Institute, Durban, Kwazulu Natal South Africa; 13 Department of Medicine, College of Medicine, University of Ibadan Oyo State, Ibadan, Nigeria; 14 Hong Kong Jockey Club, Global Health Institute, Hong Kong SAR, China

Correction to: *Scientific Reports* 10.1038/s41598-025-06536-2, published online 29 July 2025

The original version of this Article contained an error in the indexing of the name of the author Mazharul Altaf, which was incorrectly stated as Altaf Mazharul.

The original Article has been corrected.

