# Supplementary material for: Safety and immunogenicity of fractional COVID-19 vaccine doses in Nigerian adults: A randomized non-inferiority trial
Source: Sci Rep. 2025 Jul 29;15:27614. doi: 10.1038/s41598-025-06536-2 (PMC12307607; doi:10.1038/s41598-025-06536-2)
Supplement: Supplementary file 1 — Supplementary Information. [file 41598_2025_6536_MOESM1_ESM.pdf]

Supplementary Table 1. Characteristics of COVID-19 Vaccines used for the study

Type of Vaccine	Doses/Vial	Composition	Full dose	Lot Number	Expiry Date
BNT162B2	6	30µg/dose	0.3mL	FM7379	08/2022
ChadOx1	10	5×10^{10} vp/mL	0.5mL	4121Z260	07/2022
Ad26.COV2.S	5	1.0×10^{11}vp/mL	0.5mL	ACC5333	10/2023

Supplementary Table 2. Baseline Characteristics of the Study Population based on the Primary Objective based on Vaccine dose (n= 540)

	**Dose (n= 540)**		
**Variables**	**Quarter**	**Half**	**Full**
**Mean age (± SD) years**	35.3 ± 12.8	35.8 ± 12.9	38.25 ± 13.5
**Age Group**			
18-55 years<br>56-65 years	114(91.9)<br>10(8.1)	182(92.9)<br>14(7.1)	194(88.2)<br>26(11.8)
**Sex**			
Female	79(63.7)	111(56.6)	132(60)
Male	45(36.3)	85(43.4)	88(40)
**Marital Status**			

Married	70(56.5)	101(51.5)	120(54.5)
Unmarried	54(43.5)	95(48.5)	100(45.5)
**Educational Level**			
None	12(19.7)	10(5.1)	13(5.9)
Primary	19(15.3)	35(17.9)	36(16.4)
Secondary	58(46.8)	97(49.5)	119(54.1)
Tertiary	35(28.2)	54(27.6)	52(23.6)
Occupation			
Employed	92(74.2)	151(77)	174(79.1)
Unemployed	32(25.8)	45(23)	46(20.9)
**Average Income**			
1-30,000	66(53.2)	100(51)	99(45)
Above 30,000	22(17.7)	35(17.9)	48(21.8)
**Seropositivity at baseline**			
**Negative**	36(30.6)	66(33.7)	70(31.8)
**Positive**	86(69.4)	129865.8)	150(68.2)

Supplementary Table 3: Adverse Events among the study participants by Vaccine type and Dose

Adverse events (present)	ChadOx1		BNT162B2		Ad26.COV2.S	
	Fractional doses (Quarter/Half)	Full	Fractional doses (Half)	Full	Fractional doses (Quarter/Half)	Full
Pain at injection site	27 (43.5%)	24 (38.7%)	6 (16.7%)	16 (44.4)	22 (28.6%)	32 (41.6)
Heavy arm	2 (3.2%)	0 (0.0%)	0 (0.0%)	2 (5.6%)	0 (0.0%)	1 (1.3%)
Itching at injection site	0 (0.0%)	4 (6.5%)	0 (0.0%)	0 (0.0%)	1 (1.3%)	0 (0.0%)
Swelling at injection site	0 (0.0%)	0 (0.0%)	1 (44.2%)	0 (0.0%)	0 (0.0%)	0 (0.0%)
Rash at injection site	0 (0.0%)	0 (0.0%)	0 (0.0%)	0 (0.0%)	0 (0.0%)	0 (0.0%)
Fever	5 (8.1%)	9 (14.5%)	5 (6.5%)	7 (9.1%)	2 (5.6%)	4 (11.1%)
Chills and Rigor	0 (0.0%)	1 (1.6%)	0 (0.0%)	0 (0.0%)	0 (0.0%)	0 (0.0%)
Nausea	0 (0.0%)	0 (0.0%)	0 (0.0%)	0 (0.0%)	0 (0.0%)	0 (0.0%)
Body aches	2 (3.2%)	1 (1.6%)	3 (8.3%)	3 (8.3%)	6 (7.8%)	5 (6.5%)
Headaches	2 (3.2%)	4 (6.5%)	3 (8.3%)	2 (5.6%)	2 (2.6%)	2 (2.6%)
Insomnia (unable to sleep)	0 (0.0%)	1 (1.6%)	0 (0.0%)	0 (0.0%)	0 (0.0%)	0 (0.0%)
Eye pain	0 (0.0%)	0 (0.0%)	0 (0.0%)	0 (0.0%)	1 (1.3%)	0 (0.0%)
Fatigue	7 (11.3%)	10 (16.1%)	4 (11.1%)	1 (2.8%)	4 (5.2%)	4 (5.2%)
Nasal discharge	0 (0.0%)	0 (0.0%)	1 (2.8%)	0 (0.0%)	1 (1.3%)	0 (0.0%)

Cough	0 (0.0%)	0 (0.0%)	0 (0.0%)	1 (2.8%)	2 (2.6%)	1 (1.3%)
Difficulty in breathing	0 (0.0%)	0 (0.0%)	0 (0.0%)	0 (0.0%)	0 (0.0%)	0 (0.0%)
Seizures	0 (0.0%)	0 (0.0%)	0 (0.0%)	0 (0.0%)	0 (0.0%)	0 (0.0%)
Loss of consciousness	0 (0.0%)	0 (0.0%)	0 (0.0%)	0 (0.0%)	0 (0.0%)	0 (0.0%)
Passage of watery stools	0 (0.0%)	0 (0.0%)	0 (0.0%)	0 (0.0%)	0 (0.0%)	0 (0.0%)
Vomiting	0 (0.0%)	0 (0.0%)	0 (0.0%)	0 (0.0%)	1 (1.3%)	0 (0.0%)
Redness of eye	0 (0.0%)	0 (0.0%)	0 (0.0%)	0 (0.0%)	0 (0.0%)	0 (0.0%)
Increased appetite	1 (1.6%)	0 (0.0%)	1 (2.8%)	1 (2.8%)	1 (1.3%)	1 (1.3%)
Slept a lot	0 (0.0%)	0 (0.0%)	1 (2.8%)	1 (2.8%)	1 (1.3%)	2 (2.6%)

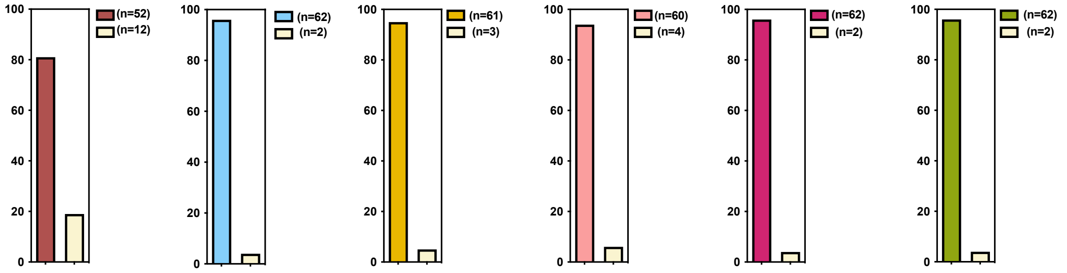

Supplementary Figure 1: Luminex analysis of serum binding antibodies in a subset of participants
Proportion of participants who were positive and negative for total IgG antibodies against SARS COV-2 Wu-1 anti-Nucleocapsid against SARS COV-2 Wu-1 anti-Nucleocapsid (anti-N), anti-Spike (S), anti-Receptor Binding Domain (Wu-1 RBD), anti-Receptor Binding Domain (Omicron BA.1 RBD), anti-Spike-1 (S1) and anti-Spike-1 Omicron (S1 Omicron) antibodies based on total number of participants recruited with samples available across three timepoints (n=64).

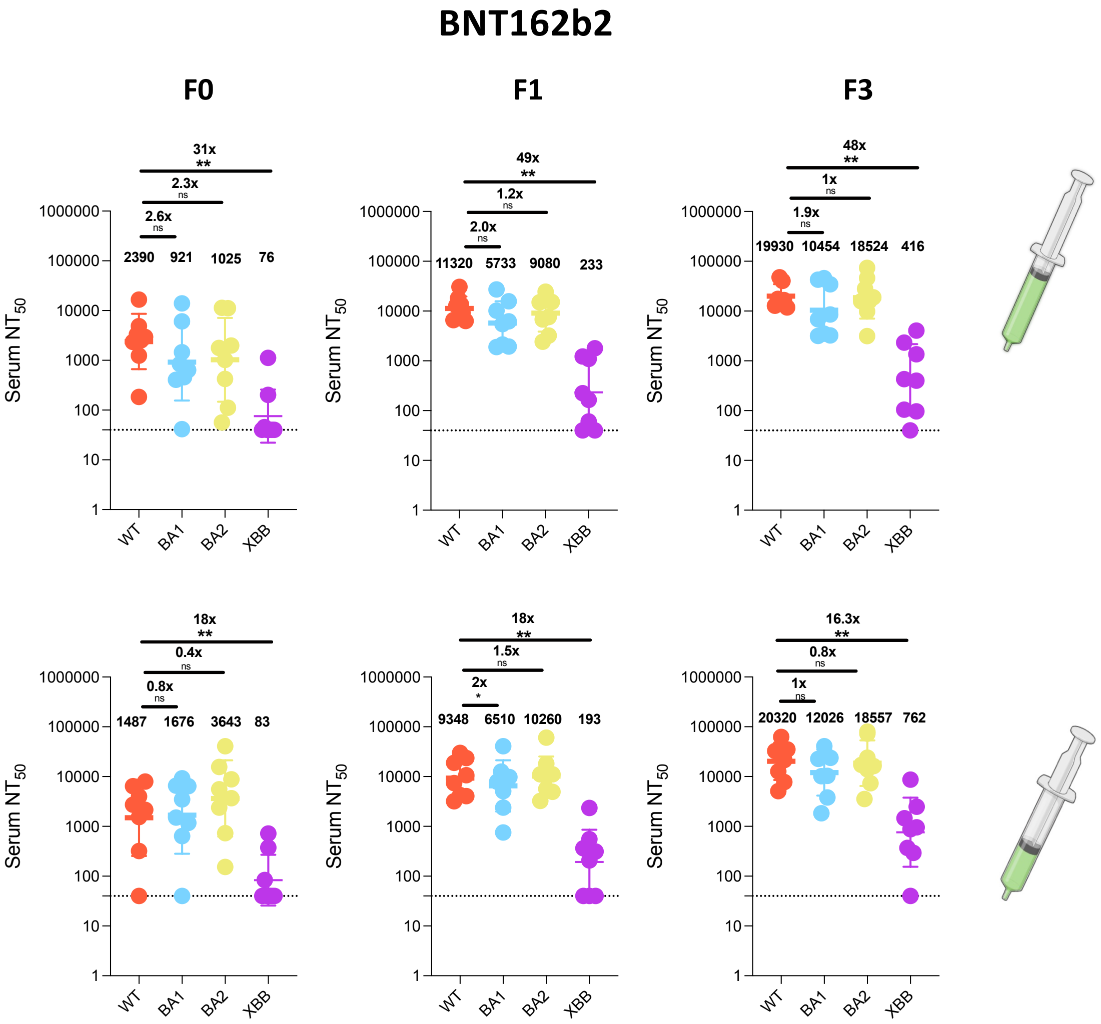

Supplementary Figure 2: Antibody responses to vaccination with BNT162b2 at full and half doses. a): Plasma neutralization of pseudovirus against Wild type (Wu-1), BA.1, BA.2 and XBB after two doses of the **BNT162b2** in Nigerian HIV-negative participants in Lagos Nigeria at three consecutive time points– baseline – F0 (before first-dose vaccination),F1 (1 month after 1st dose vaccination) and F5 (1-month post second dose at full doses (n=8) and half doses (n=8).

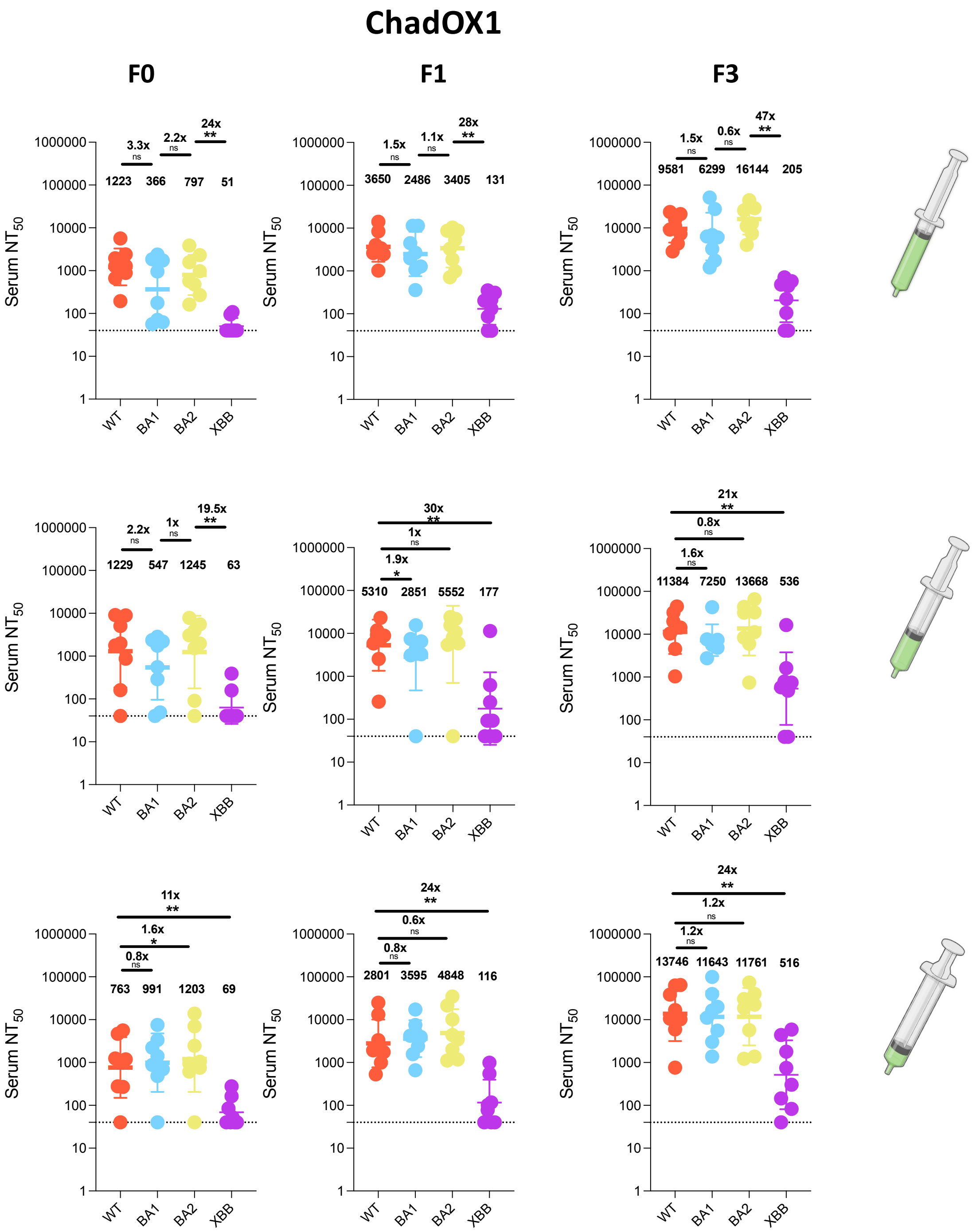

Supplementary Figure 3: Antibody responses to vaccination with ChadOX1 at full, half and quarter doses.

a): Plasma neutralization of pseudovirus against Wild type (Wu-1), BA.1, BA.2 and XBB after two doses of the **ChadOX1** in Nigerian HIV-negative participants in Lagos Nigeria at three consecutive timepoints– baseline – F0 (before first-dose vaccination), F1 (1 month after 1st dose vaccination) and F5(1-month post second dose at full, (n=8); half (n=8) and quarter doses (n=8).

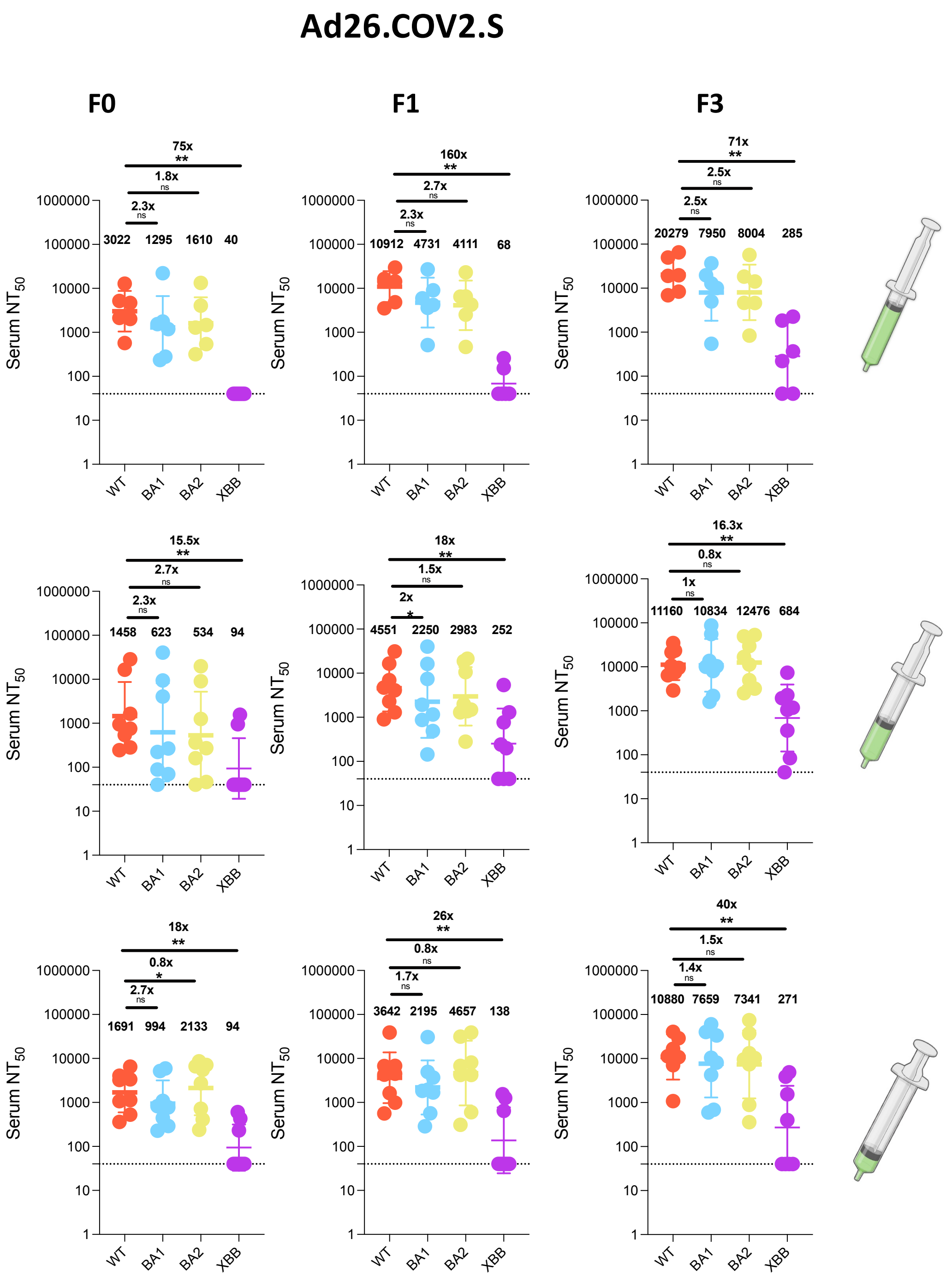

Supplementary Figure 4 : Antibody responses to vaccination with Ad26.COV2.S at full, half and quarter doses.

a): Plasma neutralization of pseudovirus against Wild type (Wu-1), BA.1, BA.2 and XBB after two doses of the Ad26.COV2.S in Nigerian HIV-negative participants in Lagos Nigeria at three consecutive time points– baseline – F0 (before first-dose vaccination), F1 (1 month after 1st dose vaccination) and F5 (1-month post second dose at full, (n=6); half (n=8) and quarter doses (n=8).
